# Effectiveness of long-lasting insecticidal nets for malaria elimination in Laos (2016-2023)

**DOI:** 10.5281/zenodo.15479515

**Published:** 2025-05-21

**Authors:** Nouanthong Navalith, Heon Jae Jeong, Yeun Soo Yang, Nouanthong Phonethipsavanh, Sangyune Kim, Sunjoo Kang

**Affiliations:** 1 Graduate School of Public Health, Yonsei University, Seoul, Republic of Korea.; 2 The Care Quality Institute, Seoul, Republic of Korea.; 3 Institute for Health Promotion, GSPH, Yonsei University, Seoul, Korea.; 4 Department of Epidemiology and Health Promotion, GSPH, Yonsei University, Seoul, Korea.; 5 Institute Pasteur du Laos, Ministry of Health, Vientiane, Lao PDR.

## Abstract

**Introduction:**

Malaria remains a significant health challenge in Laos, particularly in the southern provinces with dense forests and mobile populations. Despite progress in reducing cases, socio-environmental factors drive its persistence.

**Materials and Methods:**

Using data from 2016 to 2023, trends were analysed with P-trend analysis, and effects of long-lasting insecticidal nets (LLIN) and climate on malaria incidence were assessed via Poisson regression.

**Results:**

During this period, malaria incidence decreased by 95.5%, underscoring the success of elimination strategies. LLIN distribution led to a 54.1% reduction in incidence (IRR=0.459; p 0.002). Climate factors did not significantly influence transmission rates (IRR=0.67; p 0.717).

**Conclusions:**

The critical role of LLINs in reducing malaria incidence is evident. To support the national elimination goal for 2030, interventions must maintain consistent coverage and community engagement. Future research should focus on localised climatic data and address specific challenges in regions like Khammouane Province, enhancing the effectiveness of malaria control programmes and improving intervention strategies.

## Introduction

Malaria remains a major global health concern, with an estimated 263 million cases and 597,000 deaths reported worldwide in 2023, showing an increase of 11 million cases from the previous year. [1]. Despite significant global progress (a 27% decline in incidence and 51% reduction in mortality between 2000 and 2020) the disease persists in tropical and subtropical regions, with sub-Saharan Africa and Southeast Asia bearing the greatest burden due to complex socio-economic, geographical, and environmental factors [2].

In Southeast Asia, the Greater Mekong Subregion (GMS)—comprising Cambodia, China's Yunnan Province, Laos, Myanmar, Thailand, and Vietnam—has emerged as a focal point for malaria elimination. Comprehensive interventions, including the distribution of long-lasting insecticidal nets (LLINs), indoor residual spraying, and artemisinin-based combination therapy (ACT), have significantly reduced malaria incidence across the region. Notably, China achieved malaria elimination in 2021, setting a benchmark for other countries in the GMS [2,3].

In Laos, malaria cases decreased from 22,788 in 2009 to 695 in 2023, largely due to targeted vector control strategies, active case detection, and the integration of surveillance systems such as DHIS2 [4,5]. However, malaria remains endemic in the southern provinces, where dense forests, mobile populations, and limited healthcare access pose persistent challenges. Environmental factors, such as deforestation and seasonal rainfall, exacerbate malaria transmission by influencing vector breeding habitats [6,7]. Socio-economic barriers, including inconsistent intervention coverage and limited community engagement, further hinder malaria control efforts in these high-burden regions [8].

Although LLINs are widely recognised as a cornerstone of malaria prevention, there is limited evidence evaluating their effectiveness under varying climatic and socio-environmental conditions in high-burden settings like Laos. This gap underscores the need for localised evaluations to guide intervention strategies effectively [9-11].

This study aimed to address these gaps by evaluating the effectiveness of LLIN distribution in reducing malaria incidence and examining the influence of climate variability on malaria transmission in Laos. The findings aim to provide evidence-based insights aligned with the Lao National Strategic Plan (2016–2025) to support the country’s goal to eliminate malaria by 2030 [5].

## Materials and Methods

This retrospective, quantitative study utilised secondary data from the DHIS2 system, the central repository for malaria incidence, intervention coverage, and epidemiological indicators from Laos. Data were collected from all provinces between 2016 and 2023, focusing on high-burden malaria areas identified by the Center of Malariology, Parasitology, and Entomology (CMPE) under the Lao Ministry of Public Health. The dataset included annual malaria case counts, the distribution of LLINs, and provincial climate classifications [4,5].

### Variable definitions and operationalisation

The key variables that were incorporated in the analysis included malaria incidence, LLIN distribution, climate classification, and year (Table 1).

**Table 1:** Summary of variables and descriptions.

Variable	Type	Description
Malaria incidence	Dependent	Annual count of reported malaria cases per province. Represents the number of new cases each year in each province.
Distribution of LLIN status	Independent	For Poisson analysis: Binary indicator of LLIN distribution coverage each year per province (1 = covered, 0 = not covered). For P-trend analysis: Annual percentage of the target population covered by LLINs in each province.
Climate	Independent	A categorical variable reflecting provincial climate classification, coded as: 1: Tropical Monsoon Climate (Aw) 2: Subtropical Highland Climate (Cwb)
Year	Factor	Categorical representation of each study year from 2016 to 2023. These variables capture temporal trends in malaria incidence and LLIN interventions.

### Climate factors

While the primary climate consideration was the provincial climate classification (Aw or Cwb), this served as a proxy for broader environmental conditions that influenced vector ecology. If data had been available, analyses could have incorporated more granular climatic indicators (e.g., rainfall and temperature); however, this study used climate classification as a key independent variable.

### Statistical analysis

All statistical analyses were conducted using Python (statsmodels library) and RStudio (Version 2024.09.0).

Temporal changes in malaria incidence and LLIN coverage were characterised using descriptive statistics, including mean, median, and standard deviation. A P-trend analysis was performed using linear regression, treating year as a continuous variable and malaria incidence as the dependent variable. Statistical significance was set at p< 0.05 [5,6].

Poisson regression was applied to assess the impact of LLIN distribution and climate classification on malaria incidence. This model is appropriate for count data and assumes malaria cases follow a Poisson distribution. The model included a province-specific random effect for unobserved heterogeneity among provinces. Results were presented as incidence rate ratios (IRRs) with 95% confidence intervals (CIs). An IRR<1 indicated a reduction in malaria incidence associated with the independent variable [10,11].

Provincial climate classifications served as a proxy for broader environmental conditions influencing malaria transmission. While granular climatic data (e.g., rainfall, temperature) were unavailable, the Köppen classification provided a robust framework for analysing macro-level trends [7,9].

### Ethical approval

Ethical approval was not required for this study, as it used anonymised secondary data provided by CMPE. No direct interaction with participants or collection of personally identifiable information occurred. Approval for data use was obtained from the Department of Communicable Disease Control, Lao Ministry of Public Health (Approval No. 943, dated 25-10-2024).

## Results

**Figure 1:**
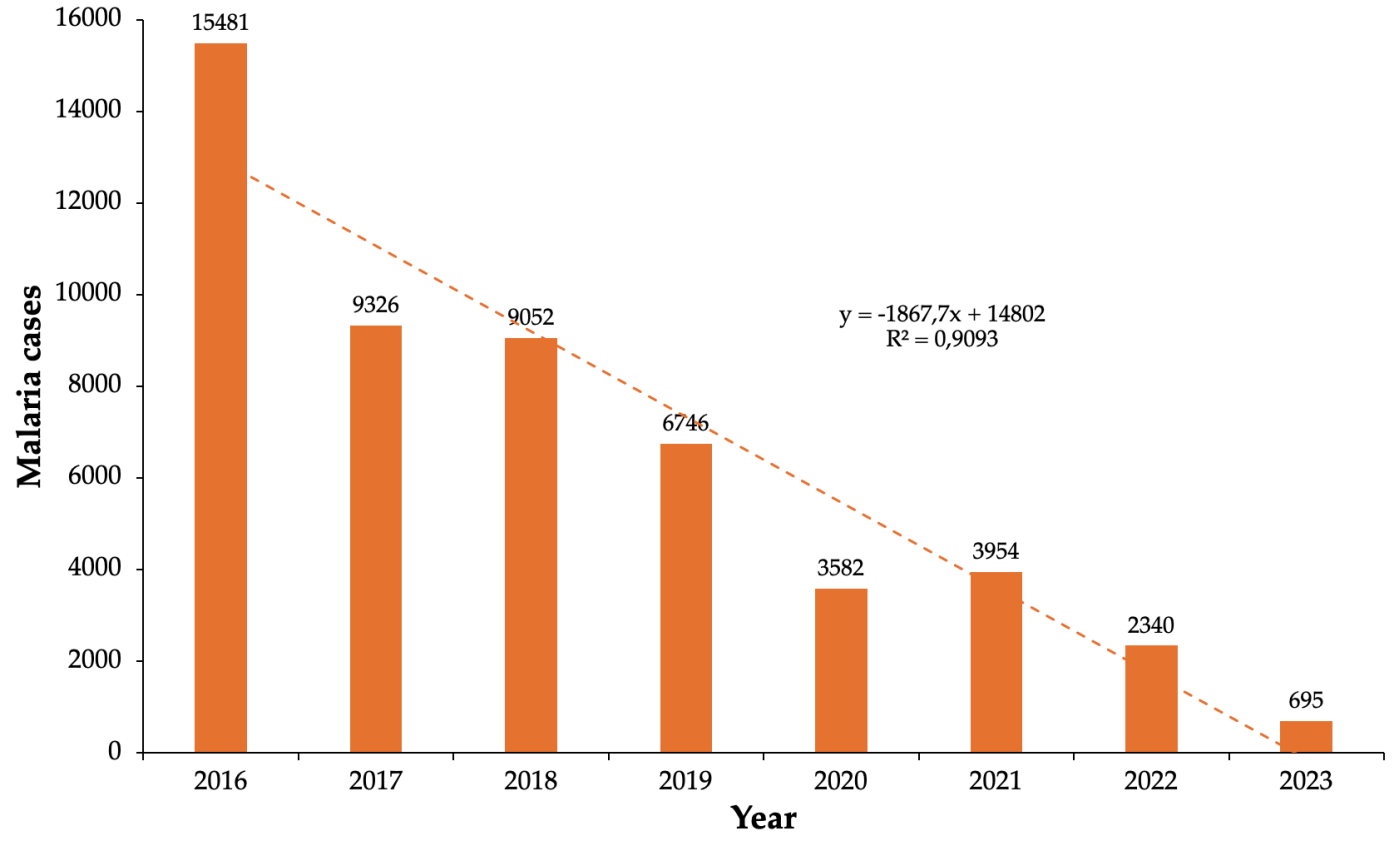
Reported malaria cases by year over the period 2016-2023. Dotted line shows the significant linear trend.

Between 2016 and 2023, malaria cases in Laos decreased by 95.5%, from 15,481 to 695 (p<0.001), demonstrating the success of elimination strategies. Over half of the provinces (55.6%) showed statistically significant reductions, while 38.9% had non-significant downward trends. One province (Khammouane) experienced increased malaria cases, likely due to intervention gaps or improper LLIN usage (Table 2, Figure 1).

**Table 2:** Trend analysis of malaria cases in Laos (2016-2023). Data was collected from the CMPE, DCDC, Laos MoH (P trend analysis).

Provience	Year (case)									Trend
	2016	2017	2018	2019	2020	2021	2022	2023	P	
Vientiane Capital	49	23	9	8	2	2	5	2	0.018	*Decrease*
Phongsaly	428	147	82	3	0	0	0	0	0.024	*Decrease*
Luang Namtha	16	12	1	2	1	0	0	0	0.014	*Decrease*
Oudomxay	55	22	6	0	0	0	1	0	0.029	*Decrease*
Bokeo	5	0	0	0	0	0	0	0	0.134	Decrease
Luang Prabang	346	55	9	10	25	2	0	0	0.072	Decrease
Houaphanh	97	1	0	2	2	0	0	0	0.127	Decrease
Xayabouly	149	15	8	3	5	0	1	0	0.084	Decrease
Xiangkhouang	11	2	1	1	0	3	2	0	0.127	Decrease
Vientiane Province	42	13	5	2	1	1	0	0	0.031	*Decrease*
Borikhamxay	32	12	10	1	3	2	2	0	0.016	*Decrease*
Khammouane	114	124	139	48	187	145	39	250	0.487	Increase
Savannakhet	1471	2376	2250	1344	406	850	244	53	0.008	*Decrease*
Saravan	3412	1949	1529	1570	317	511	464	70	0.001	*Decrease*
Sekong	1771	1175	973	1155	882	780	380	112	0.000	*Decrease*
Champasak	6214	2273	1854	909	170	141	143	37	0.011	*Decrease*
Attapeu	1215	1083	2172	1684	1581	1516	1059	171	0.239	Decrease
Xaisomboun	54	44	4	4	0	1	0	0	0.017	*Decrease*
Total	15481	9326	9052	6746	3582	3954	2340	695	0.000	*Decrease*

National LLIN coverage remained high, averaging 92.5% and improving from 89.9% in 2019 to 99.5% by 2022–2023 (p=0.224). However, certain provinces showed fluctuations or declines, highlighting logistical challenges (Table 3).

**Table 3:** Trend analysis of LLIN distribution in the high-burden areas of Laos. Data was collected from the CMPE, DCDC, Laos MoH (P trend analysis).

Province	Year/LLIN coverage (%) in target population)					Mean (%)	Std (%)	P	Trend
	2019	2020	2021	2022	2023				
Phongsaly	99.4	99.4	99.4	85.55	85.55	93.86	6.785	0.058	Decrease
Luang Namtha	0	0	10.6	10.6	10.6	6.36	5.81	0.058	Increase
Oudomxay	100	100	100	0	0	60	48.990	0.058	Decrease
Luang Prabang	83.22	83.22	83.22	104	104	91.532	10.180	0.058	Increase
Khammouane	106.45	106.45	106.45	100	100	103.87	3.160	0.058	Decrease
Savannakhet	95.53	95.53	95.53	97.12	97.12	96.166	0.779	0.058	Increase
Saravan	99.52	99.52	99.52	98.7	98.7	99.192	0.402	0.058	Decrease
Sekong	93.14	93.14	93.14	100	100	95.884	3.361	0.058	Increase
Champasak	97.77	97.77	97.77	101.06	101.06	99.086	1.612	0.058	Increase
Attapeu	99.81	99.81	99.81	100	100	99.886	0.093	0.058	Increase
Xaisomboun	24.39	24.39	24.39	99.78	99.78	54.546	36.933	0.058	Increase
Average	89.92	89.92	83.67	99.53	99.53	92.51	6.89	0.215	Decrease
# of Provinces	10	10	11	10	10				

### Effectiveness of LLIN distribution in high-burden areas on malaria incidence

Poisson regression analysis revealed that LLIN distribution was associated with a 54.1% reduction in malaria incidence (IRR = 0.4594; 95% CI: 0.2816– 0.7495; p = 0.002), underscoring its critical role in reducing transmission (Table 4).

**Table 4:** Impact analysis of LLIN distribution and climate types on malaria incidence (2016-2023).

Variable	IRR	Sth. Err.	Z	P>|z|	95% Cl
LLIN Distribution	0.4594	0.1147	-3.11	0.002	0.2816 - 0.7495
Cwb*	0.6702	0.7402	-0.36	0.717	0.0769 - 5.8390
Constant	162.4744	87.8022	9.42	0.000	56.3365 - 468.5756
Year 2017	0.3991	0.0596	-6.14	0.000	0.2977 - 0.5350
Year 2018	0.2338	0.0698	-4.87	0.000	0.1302 - 0.4198
Year 2019	0.2117	0.1016	-3.23	0.001	0.0825 - 0.5427
Year 2020	0.0592	0.0363	-4.61	0.000	0.0178 - 0.1971
Year 2021	0.0327	0.0246	-4.54	0.000	0.0074 - 0.1431
Year 2022	0.0093	0.0083	-5.23	0.000	0.0016 - 0.0538
Year 2023	0.0012	0.0013	-6.4	0.000	0.0001 - 0.0098

* = Climate (type 2)/Subtropical Highland Climate

### Mixed-effects Poisson regression analysis

144 Observations across 18 groups (regions) with 8 observations for each group (min=8, avg 8.0, max= 8); model specifications include 7 integration points, Wald chi-squared (9)=2398.98, log-likelihood= -2097.1772, and Prob > chi-squared = 0.0000.

### Impact of climate type on malaria

Climate classification, categorised as Tropical Monsoon (Aw) or Subtropical Highland (Cwb), did not exhibit a statistically significant effect on malaria incidence (IRR=0.67; 95% CI: 0.08–5.84; p 0.717). The high LLIN coverage in most provinces likely mitigated the influence of climate on malaria transmission.

In Tropical Monsoon zones, malaria cases declined significantly from 14,891 in 2016 to 695 in 2023 (p< 0.001), highlighting the effectiveness of intervention efforts in high-burden areas. Similarly, in subtropical highland zones, malaria cases decreased from 590 in 2016 to zero by 2023 (p=0.028). The rapid elimination in these less endemic areas reflects the success of highly focused interventions and smaller at-risk populations (Table 5).

**Table 5:** Malaria cases by climate zones in Laos (2016–2023) with statistical trends.

Year	Total (cases)	Tropical monsoon climate (Aw)	Subtropical highland climate (Cwb)
2016	15,481	14,891	590
2017	9,326	9,132	194
2018	9,052	8,965	87
2019	6,746	6,736	10
2020	3,582	3,580	2
2021	3,954	3,950	4
2022	2,340	2,338	2
2023	695	695	0
P-value	0	0	0.028
Trend	Decrease	Decrease	Decrease

### Regional disparities

Khammouane Province exhibited a notable increase in malaria incidence despite consistently high LLIN coverage (>100%). This anomaly suggests challenges such as insecticide resistance, improper net usage, or environmental factors. Addressing these issues requires alternative vector control strategies and enhanced community engagement to promote proper net usage and adherence to intervention protocols.

## Discussion

### Malaria incidence trends in Laos (2016-2023)

The significant decline in malaria incidence across Laos (p<0.001) from 2016 to 2023 underscores the sustained effectiveness of national malaria control interventions. Over this period, cases decreased by 95.5%, aligning with similar findings from longitudinal studies [12,13]. This reduction reflects the structured implementation of the Malaria National Strategic Plans (2016–2020 and 2021–2025), with Phase 1 targeting high-burden areas and Phase 2 expanding efforts toward nationwide elimination [5].

Despite this progress, 38.9% of provinces exhibited non-significant downward trends, likely due to inconsistent intervention coverage, environmental variability, or limited adherence to vector control measures. Khammouane Province, notably, showed an increase in malaria cases despite high LLIN coverage (>100%). This anomaly may stem from challenges such as insecticide resistance, improper net usage, or specific environmental conditions. Similar trends have been observed in Tanzania, highlighting the need for robust community engagement, alternative vector control strategies, and environmental management [14].

### Effectiveness of LLINs

The Poisson regression analysis demonstrated a 54.1% reduction in malaria incidence associated with LLIN distribution (IRR=0.459; 95% CI: 0.282–0.750; p 0.002). This finding underscores the critical role of LLINs as a cornerstone of malaria prevention efforts, particularly in high-burden regions. Consistent coverage rates, averaging 92.51% and exceeding 97% in targeted populations, were key to this success.

Southern provinces such as Savannakhet, Saravan, Sekong, and Champasak benefited from well-executed distribution campaigns. However, provinces like Oudomxay and Phongsaly experienced coverage fluctuations after 2021, highlighting logistical challenges and barriers to access. Evidence from Ethiopia and Ghana demonstrates that community engagement and education are essential to overcoming such obstacles and maximising LLIN utilisation [15,16].

Sociodemographic disparities in LLIN usage further emphasise the need for tailored interventions. As observed in Ghana, urban-rural differences in net usage stress the importance of addressing local behavioural and cultural factors. Strengthening community education alongside distribution campaigns can promote consistent and proper usage, enhancing the overall impact of LLINs [17].

### Impact of climate on malaria

The study found no statistically significant association between provincial climate types (Tropical Monsoon vs. Subtropical Highland) and malaria incidence (IRR 0.67; p 0.717). This finding suggests that high LLIN coverage may mitigate the effects of climatic factors. However, broad Köppen climate classifications may have limited the analysis. Previous studies have demonstrated that localised climatic variables, such as rainfall, humidity, and temperature variability, significantly influence malaria dynamics [9,18].

Further research incorporating granular climatic data is essential to understand the nuanced relationships between environmental factors and malaria transmission. These insights could guide adaptive vector control strategies, especially with fluctuating transmission patterns.

### Donor transition and sustainability

Sustaining malaria control efforts in Laos requires effective donor transition policies. Evidence from global health financing highlights the importance of clearly articulated long-term visions, explicit transition policies, and coordinated donor efforts. Key Asian Pacific donors, such as the Global Fund, Gavi, the World Bank, and USAID, have emphasised these principles, but their success depends on local adaptation and implementation [19].

To ensure sustainability, Laos must integrate lessons from successful donor transitions. This includes enhancing donor coordination, evaluating long-term impacts, and establishing domestic funding mechanisms such as a national malaria elimination fund. These measures align with global best practices and are critical to bridging funding gaps left by donor withdrawal. Securing reliable domestic resources will also foster national ownership and long-term independence in malaria control efforts.

### Policy implications

Policymakers must prioritise consistent distribution and use of insecticidal nets, especially in underserved areas. Efforts should target peak transmission seasons and incorporate community education and leadership engagement. Robust surveillance systems, improved digital platforms, and trained health workers are essential for real-time monitoring and data-driven decisions. Sustainable funding mechanisms and regional cooperation within the Greater Mekong Subregion will address mobility and drug resistance challenges. Climate adaptation should be integrated into malaria policies using predictive tools to align prevention measures.

### Study limitations

This study relied on secondary data from the DHIS2 system, with access limitations potentially affecting data quality and completeness. Variability in data collection and reporting standards introduced potential biases, limiting generalisability. The study focused solely on insecticidal nets, excluding other critical malaria elimination strategies such as community engagement and active surveillance. These limitations highlight the need for improved data access, quality assurance, and a broader analysis of interventions.

### Recommendations for future research

Future studies should explore interventions beyond insecticidal nets, such as community engagement and universal health coverage integration. Individual-level demographic and behavioural data and localised climate variables like rainfall and temperature should be incorporated. Investigating rising malaria rates in Khammouane Province and assessing insecticidal net adherence and longevity are critical. Socioeconomic and behavioural factors influencing intervention uptake must be evaluated, and sustainable funding models should be developed to support long-term national ownership of malaria control.

## Conclusions

This study demonstrates that LLIN distribution significantly reduced malaria incidence in Laos, with an Incidence Rate Ratio (IRR) of 0.4594, indicating a 54.1% reduction in malaria cases in intervention areas compared to non-intervention areas (p=0.002). Nationally, malaria cases declined by 95.5% from 2016 to 2023, highlighting the success of elimination strategies, although disparities in progress remain across provinces. The findings emphasise the critical role of consistent and equitable LLIN coverage in sustaining progress toward malaria elimination.

In contrast to the protective effect of LLINs, climatic factors such as Subtropical Highland Climate (Cwb) did not show a statistically significant association with malaria incidence (p=0.717). This suggests that future research should explore other potential environmental and socioeconomic contributors to malaria dynamics while focusing on improving intervention coverage and addressing gaps in high-burden areas like Khammouane Province. Addressing these challenges, along with enhancing data accessibility and standardisation within the DHIS2 system, will be essential to achieving Laos’ malaria elimination goals by 2030.
